# A pH-Responsive Dendritic-DNA-Based Nanohydrogel for Dual Drug Delivery

**DOI:** 10.3390/biom15040537

**Published:** 2025-04-06

**Authors:** Jing Zhao, Jingyuan Wu, Yiqi Fan, Chao Yu, Le Yu, Fangwei Shao

**Affiliations:** 1 Department of Chemistry, Zhejiang University, Hangzhou 310000, China; 11937033@zju.edu.cn (J.Z.); fanyiqi@zju.edu.cn (Y.F.); leyu@zju.edu.cn (L.Y.); 2 National Key Laboratory of Biobased Transportation Fuel Technology, Zhejiang University-University of Illinois, Urbana-Champaign Institute, Zhejiang University, Haining 314400, China; 3 Division of Chemistry and Biological Chemistry, Nanyang Technological University, Singapore 639956, Singapore; jingyuanwu@ntu.edu.sg; 4 Department of Orthopedics, 2nd Affiliated Hospital, School of Medicine, Zhejiang University, Hangzhou 310000, China; 12118514@zju.edu.cn; 5 Orthopedics Research Institute of Zhejiang University, Zhejiang University, Hangzhou 310000, China

**Keywords:** nanohydrogels, stimulus-responsive nanomaterials, targeted delivery, chemotherapy, gene silencing, dual treatments

## Abstract

The rational design of multifunctional drug delivery systems capable of achieving precise drug release remains a huge challenge. Herein, we designed a stimuli-responsive dendritic-DNA-based nanohydrogel as a nanocarrier to achieve the co-delivery of doxorubicin and *HMGN5* mRNA-targeting antisense oligonucleotides, thus achieving dual therapeutic effects. The nanocarrier, constructed from dendritic DNA with three crosslinking branches and one loading branch, formed biocompatible and programmable DNA nanohydrogels. The C-rich sequences in the crosslinking branches conferred pH sensitivity, while the loading strand enabled efficient incorporation of a shielding DNA/ASO complex. DOX encapsulation yielded a chemo–gene co-delivery platform. Upon cellular uptake by cancer cells, the nanocarrier disassembled in the acidic tumor microenvironment, releasing DOX for chemotherapy and ASOs via toehold-mediated strand displacement (TMSD) for targeted gene silencing. Cellular studies demonstrated significantly enhanced cancer cell inhibition compared to single-agent treatments, highlighting strong combined effects. This study provides a novel strategy for tumor-microenvironment-responsive co-delivery, enabling precise, on-demand release of therapeutic agents to enhance combined chemo–gene therapy.

## 1. Introduction

Cancer remains a major global health challenge, with approximately 19.3 million new cases and 10 million deaths reported in 2020, and this number is expected to rise to 28.4 million new cases annually by 2040 [1]. Nanohydrogels represent a novel class of nanomaterials characterized by their high water content and biocompatibility, emerging as effective drug delivery systems in cancer therapy [2,3]. Their hydrophilic polymer networks facilitate the encapsulation of various therapeutic agents, enhancing the solubility of hydrophobic drugs and minimizing systemic toxicity [4,5]. Specifically, DNA nanohydrogels leverage the unique structural and functional properties of DNA to provide superior programmability and biocompatibility [6,7] in order to tailor drug release. The programmability renders DNA nanohydrogels particularly suitable for the delivery of genetic therapeutics [8,9], thereby achieving dual effects [10] to expand the scopes of conventional chemo-drugs.

However, precise spatial and temporal manipulation of drug release remains a significant challenge in the development of DNA nanohydrogels. Overcoming these challenges is essential for the advancement of smart drug delivery systems that can optimize therapeutic outcomes. Currently, a variety of stimuli-responsive nanomaterials, including polymeric nanoparticles [11], liposomes [12], inorganic nanocarriers [13], and micelles [14], have been explored for drug delivery applications. These materials can react to specific stimuli such as pH, temperature, light, or biochemical signals, enabling targeted drug delivery and on-demand release, which can enhance therapeutic efficacy and reduce off-target effects [15]^.^ However, issues related to potential toxicity and intricate fabrication processes may restrict their broader applications. Recent advances in smart DNA functionalization strategies have demonstrated that intelligent design of DNA-based systems can significantly enhance drug delivery efficiency by enabling precise control over drug release kinetics and targeting specificity [16,17].

**Scheme 1:**
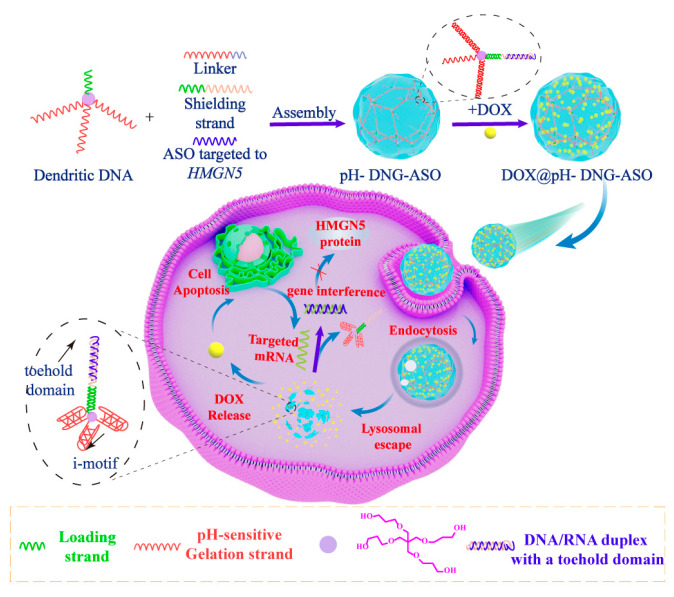
Schematic illustration of a stimuli-responsive dendritic-DNA-based nanohydrogel for dual drug delivery.

Consequently, there is an urgent need to develop DNA nanohydrogels that incorporate these responsive features. DNA nanohydrogels exhibit remarkable programmability, allowing for the design of systems responsive to specific biological stimuli, such as pH, mRNA, or specific biomolecules within tumor microenvironment [18,19]. This responsiveness would pave the way for integrating chemotherapeutics with gene therapies [20,21], aligning a timely release of therapeutic agents with targeted genetic interventions. In this context, we have developed a pH/mRNA stimuli-responsive dendritic-DNA-based nanohydrogel (**DOX@pH-DNG-ASO**) for the co-delivery of doxorubicin (DOX) and antisense oligonucleotides (ASOs) to achieve combined therapeutic effects in vitro (Scheme 1). This multifunctional DNA nanohydrogel demonstrates significant anticancer activity through a combination approach of chemo–gene treatments, which involves silencing the tumor-associated gene HMGN5 [22,23]. 

## 2. Materials and Methods

### 2.1. Chemicals and Materials

All chemicals and solvents utilized were of analytical grade. Millipore water was employed to prepare all aqueous solutions. Linear DNA and RNA strands were procured from Sangon Biotech Co., Ltd. (Shanghai, China). Modified DNA strands and all RNA strands underwent purification via high-performance liquid chromatography (HPLC). All the sequences are shown in Table S1. DEPC-treated water was used to dissolve RNA-related materials. Reagents and phosphoramidites used in solid-phase DNA syntheses were sourced from Beijing Higgrene-tech Automation Ltd. (Beijing, China). Unless otherwise specified, all chemicals were obtained from Sigma-Aldrich (St. Louis, MO, USA).

### 2.2. Oligonucleotide Synthesis

Dendritic DNA was synthesized using a solid-phase DNA synthesizer (K&A H-8, Schaafheim, Germany) with 2000 Å CPG, following our established protocol [24] and characterized via mass spectrometry (Figures S1 and S2).

### 2.3. Assembly of pH-DNG

The assembly of pH-DNG was conducted using a method similar to that of non-pH-DNG [24]. A schematic representation of the assembly process is provided in Figure S1, illustrating the key chemical routes and steps involved in the formation of pH-DNG. Dendritic DNA (100 nM) and linker (100 nM) were combined in 1× TAE/Mg^2+^ buffer (containing 40 mM Tris, 20 mM acetic acid, 2 mM EDTA, and 12.5 mM Mg^2+^, with the pH adjusted to 7.4). The mixture was heated to 95 °C and then gradually cooled to room temperature. Subsequently, the nanogels were dialyzed (using a 25K MWCO membrane from Yuanye Bio-Technology Co., Ltd., Shanghai, China) to eliminate excess dendritic DNA and linker oligonucleotides. The purified nanogels were then concentrated through lyophilization. The resulting pH-DNG powder was then diluted in 1× TAE/Mg^2+^ buffer (pH 7.4) to prepare a stock solution (Abs260 = 0.5). This stock solution was used for subsequent experiments unless otherwise indicated.

### 2.4. Particle Size and Zeta Potential Measurements

The hydrodynamic size and zeta potential of the samples were determined using a Zetasizer Advance Pro (ZX XPLORER): Version 2.3.1.4 (Malvern, UK). Each sample was measured three times to ensure accuracy.

### 2.5. Atomic Force Microscopic (AFM)

AFM images were obtained using an atomic force microscope (MFP-3D Origin+, Oxford, UK) in tapping mode at room temperature in air. The AFM measurements were performed using AC-240TSA-R3 tips (Oxford Instruments, Santa Barbara, CA, USA) with a nominal spring constant of 2 N/m and a resonance frequency of 70 kHz. Prior to data acquisition, the probes were calibrated to ensure accuracy and reproducibility. The deflection sensitivity was determined by force–distance curves on a clean silicon surface.

### 2.6. DOX Loading of pH-DNG

According to our previous work [24], doxorubicin (DOX) solution in 12.5 mM MgCl_2_, 1× TAE buffer (pH 7.4) was first shaken continuously with **pH-DNG** (0.5 OD) at room temperature for 24 h in the darkness. After the loading process, excess free DOX was removed by dialysis bag (MW = 1000 Da, Yuanye Bio-Technology Co., Ltd., Shanghai) for 24 h in the darkness. Then the concentration of DOX in the dialysate ([DOX]_out) was quantified by a UV–vis spectrometer (Shimadzu UV-2600, Kyoto, Japan), according to the extinction coefficient of DOX at 480 nm (ε DOX = 11,500). The loading number of DOX per base pair (*R*_DOX_) was calculated using the following formula:

$$R_{DOX}=([DOX{]}_{0}*V_{DOX}-[DOX{]}_{out}*V_{out})/([bp]*V_{in}),$$

where [bp] is the base pair concentration of the **pH-DNG** solution inside the dialysis bag, determined by the original OD of **pH-DNG**. The loading experiments were averaged over three repeats.

### 2.7. Doxorubicin Release

A total of 100 mL of 1× PBS buffer (pH 7.4 or 5.5) was used as drug release media. A total of 500 μL of **DOX@pH-DNG** was encapsulated within a dialysis bag (MW = 1000 Da) and stirred in 1× PBS buffer at pH 7.4 or 5.5. At the selected time intervals, 3 μL of **DOX@pH-DNG** was taken out to quantify the DOX concentration inside the dialysis membrane by UV–vis absorption spectroscopy (NanoPhotometer N60, IMPLEN, München, Germany) at 480 nm.

### 2.8. Assembly of pH-DNG-ASO

Initially, dendritic oligonucleotides (100 nM), linker strands (100 nM), shielding strands (100 nM), and ASO strands (100 nM) were mixed in 1 mL of 12.5 mM MgCl_2_, 1× TAE buffer (pH = 7.4). The mixture was heated to 95 °C and slowly cooled down to room temperature overnight. The resulting nanohydrogels were dialyzed (MW = 25,000 Da, Yuanye Bio-Technology Co., Ltd., Shanghai) to remove the excess oligonucleotides. Then, purified nanohydrogels were concentrated by lyophilization. A total of 100 μL DEPC water was added to make stock solution. Then, the stock solution was diluted to make **pH-DNG-ASO** (0.5 OD).

### 2.9. ASO Release from pH-DNG-ASO

The **pH-DNG-ASO** samples were incubated with target mRNA or scrambled RNA in 1× TAE buffer (pH 7.0) for 4 h at 37 °C. Then, the samples were characterized by 10% native polyacrylamide gel electrophoresis (PAGE) (running buffer: 1× TBE; running voltage: 350 V).

### 2.10. Doxorubicin Loading of pH-DNG-ASO

The procedure for doxorubicin loading onto **pH-DNG-ASO** is identical to that used for **pH-DNG**.

### 2.11. Cell Culture

The human breast cancer cell line MDA-MB-231 cells (catalog number TCHu227) were routinely cultured in high-glucose DMEM supplemented with 10% (*v*/*v*) fetal bovine serum (FBS) and 1% (*w*/*v*) penicillin–streptomycin under 5% CO_2_ at 37 °C. The human mammary epithelial cell line MCF-10A cells (catalog number SCSP-575) were routinely cultured in MCF-10A-specific medium under 5% CO_2_ at 37 °C. The human lung adenocarcinoma cell line A549 (catalog number: TCHu150), a model for non-small-cell lung cancer (NSCLC), was routinely cultured in RPMI-1640 supplemented with 10% (*v*/*v*) fetal bovine serum (FBS) and 1% (*w*/*v*) penicillin–streptomycin under 5% CO_2_ at 37 °C. The cancer cells were obtained from the National Collection of Authenticated Cell Cultures (NCACC).

### 2.12. Confocal Fluorescence Microscopy Imaging

MDA-MB-231 cells (2 × 10^5^ per well) were seeded in a 20 mm u-dish containing DMEM cell medium. pH-TAMRA-labeled **pH-DNG**, **DOX@pH-DNG**, Cy5-labeled **pH-DNG-ASO**, and **DOX@pH-DNG-ASO** were added to the cell medium and incubated for 4 h, respectively. Afterwards, the cell medium was removed and the cells were washed with PBS for three times. Next, the cells were fixed with 4% paraformaldehyde for 15 min and stained with DAPI according to commercial protocol. The cell medium was replaced by PBS before the confocal images were taken. All images were collected on a confocal microscope (Zeiss LSM 880) with a 40 × oil immersion objective. For DOX and TAMRA, a 488 nm laser was used for excitation, with an emission range of 580 ± 20 nm. For Cy5.5, a 633 nm laser was used for excitation, with an emission range of 668 ± 20 nm.

### 2.13. Flow Cytometry

MDA-MB-231 cells (8 × 10^5^ per well) were plated in 35 mm cell culture dishes and incubated overnight. The cells were then incubated with free DOX, **DOX@pH-DNG**, **pH-DNG-ASO**, and naked ASOs for 4 h, respectively. Following incubation, the cells were finally suspended in 400 μL PBS for flow cytometry analysis using a NovoCyte instrument (ACEA, San Diego, CA, USA). The untreated cells were used as negative control.

### 2.14. Lysosomal Colocalization of pH-DNG-ASO

MDA-MB-231 cells were seeded into confocal dishes (2 × 10^5^ cells per well) and incubated with Cy5-labeled **pH-DNG-ASO** for different durations (4 and 12 h). The cells were stained with LysoTracker Green for 2 h and Hoechst 33342 for 15 min. Then, the cells were washed three times with PBS and imaged by CLSM (LSM 880, Zeiss, Oberkochen, Germany). For LysoTracker Green, the excitation wavelength was 488 nm, and the emission range was 500–550 nm.

### 2.15. Cell Viability Assay

The MDA-MB-231 cells were seeded at a density of 1.0 × 10^4^ cells per well into the 96-well plates and cultured overnight. Then, the cells were, respectively, incubated with **pH-DNG**, free DOX, **DOX@pH-DNG**, **DOX@non-pH-DNG**, **pH-DNG-ASO**, and **DOX@pH-DNG-ASO** at different concentrations for 48 h. After incubation, CCK-8 solution was added to each group for continued culturing for 1 h. The absorption at 450 nm was measured using a microplate reader (SPARK, Tecan, Mount Waverley, Australia).

The A549 cells were seeded at a density of 1.0 × 10^4^ cells per well into the 96-well plates and cultured overnight. Then, the cells were, respectively, incubated with free DOX, **DOX@non-pH-DNG**, and **DOX@pH-DNG** at different DOX concentrations for 48 h. After incubation, CCK-8 solution was added to each group for continued culture for 1 h. The absorption at 450 nm was measured using a microplate reader (SPARK, Tecan, Australia).

### 2.16. Scratch Healing Assay

MDA-MB-231 cells were seeded into 6-well plates (5 × 10^5^ cells per well) and cultured at 37 °C overnight. A scratch was then made through the cell layer using a pipette tip along a straight line guided by a ruler. After scratching, the non-adherent cells were removed by washing with PBS three times. The cells were incubated with PBS, **pH-DNG** (0.5 OD), and **pH-DNG-ASO** (60 nM) for 0 and 24 h, respectively. Then, the change in the gap area was observed under a microscope (Leica, Wetzlar, Germany).

### 2.17. Quantitative Reverse Transcription Polymerase Chain Reaction (qRT-PCR)

The MDA-MB-231 cells were seeded into the six-well plates (2 × 10^5^ cells per well) and incubated with PBS and **pH-DNG-ASO** for 24 and 48 h, respectively. Then, the cells were washed three times with PBS. Total mRNA was extracted with Trizol agent according to the manufacturer’s instructions. Then the equal amounts of RNA were reverse-transcribed to complementary DNA (cDNA) by using a commercial kit. After that, the PCR process was carried out by mixing primers, cDNA, dNTP, and DNA polymerase. Glyceraldehyde-3-phosphate dehydrogenase (GAPDH) was amplified as an internal control. The primer sequences are shown in Table S1.

### 2.18. Western Blot

Cells were lysed using RIPA buffer supplemented with 1× protease inhibitor cocktail and 1 mM PMSF. The lysates were centrifuged at 12,000× *g* for 15 min at 4 °C to remove cellular debris. The supernatant was collected, and protein concentrations were determined using a BCA protein assay kit (Beyotime, Shanghai, China, Cat No. P0010S) according to the manufacturer’s instructions. Equal amounts of protein (20 µg per sample) were heated at 100 °C for 5 min to denature the proteins. Samples were then loaded onto 4–12% Bis-Tris precast gels alongside a pre-stained protein marker (6.5–270 kDa). Electrophoresis was performed using MOPS running buffer at 120 V for approximately 90 min or until the dye front reached the bottom of the gel. Proteins were then transferred from the gel to a PVDF membrane (Millipore, St. Louis, MO, USA, Cat No. IPVH00010) using a wet transfer system (Bio-Rad) at 100 V for 90 min in transfer buffer containing 25 mM Tris, 192 mM glycine, and 20% methanol. The membrane was blocked with 5% non-fat dry milk (Bio-Rad, Singapore, Cat No. 1706404) in TBST (20 mM Tris, 150 mM NaCl, 0.1% Tween-20, pH 7.6) for 1 h at room temperature to prevent non-specific binding. After blocking, the membrane was incubated overnight at 4 °C with the primary antibody against HMGN5 (NSBP1 Polyclonal Antibody, Proteintech, Singapore, Cat No. 23955-1-AP, 1:1000 dilution in 5% BSA/TBST). Following primary antibody incubation, the membrane was washed three times with TBST (10 min each) and incubated with a horseradish peroxidase (HRP)-conjugated secondary antibody (anti-rabbit IgG, Cell Signaling Technology, Cat No. 7074S, 1:5000 dilution in 5% non-fat dry milk/TBST) for 1 h at room temperature. After washing the membrane three times with TBST (10 min each), protein bands were visualized using an enhanced chemiluminescence (ECL) detection kit (Thermo Fisher Scientific, Singapore, Cat No. 32106) according to the manufacturer’s instructions. Images were captured using Odyssey^®^ Fc (Licor, Bourne, MA, USA). GAPDH (Cell Signaling Technology, Singapore, Cat No. 5174S, 1:5000 dilution) was used as a loading control.

### 2.19. Statistical Analysis

Statistical analysis results are presented as means ± standard deviation. Data were analyzed using one-way analysis of variance (ANOVA) in GraphPad Prism 8.0 for comparisons involving more than two groups. Statistical significance was recognized at *p* < 0.05. Asterisks represent significant differences (* *p* < 0.05, ** *p* < 0.01, *** *p* < 0.001, **** *p* < 0.0001).

### 2.20. Software Tools

In this study, multiple software tools were employed for data processing: AFM image data were processed using ImageJ software, which enabled operations such as length analysis to determine the size of nanohydrogels. DLS data were processed using Zetasizer software to analyze the particle size distribution and zeta potential of nanohydrogels. Confocal fluorescence microscopy images were acquired and initially processed using ZEN software (Version 3.4.91.00000, integrated with Zeiss LSM 880), with further colocalization analysis performed using ImageJ software. Data plotting was performed using OriginPro (Version 2021 (9.8.0.200)), while statistical analyses were conducted using GraphPad Prism.

## 3. Results

### 3.1. Design, Characterization, and Functional Evaluation of pH-Responsive DNA Nanohydrogels for Targeted Drug and Gene Delivery

#### 3.1.1. Assembly and Characterization of pH-Responsive DNA Nanohydrogels (pH-DNG)

**Figure 1:**
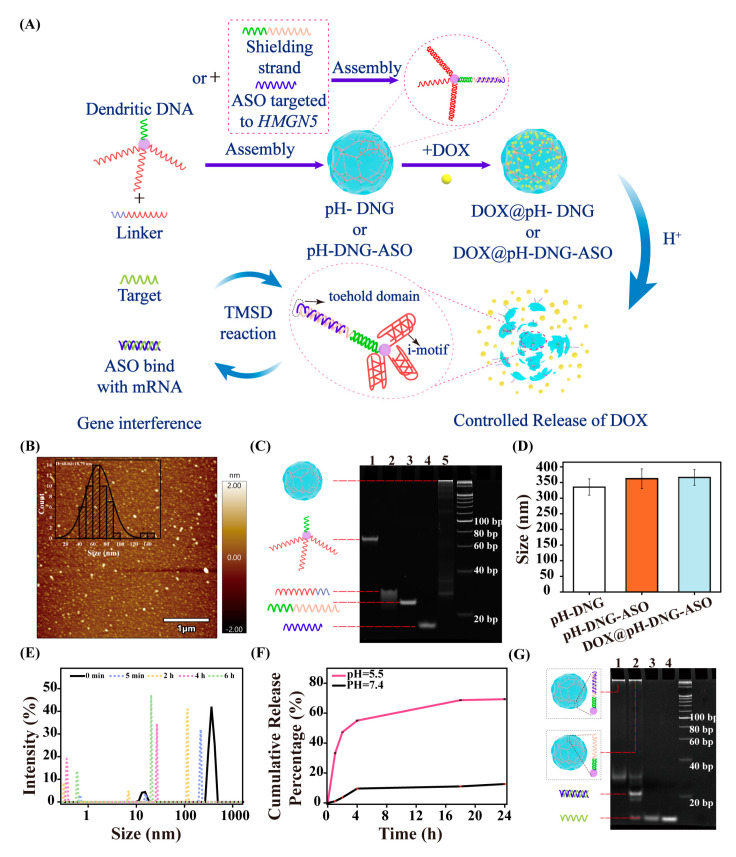
(**A**) Scheme of the construction of **DOX@pH-DNG-ASO** and the process of DOX and ASO release by responding to the stimulus. (**B**) AFM image of **pH-DNG** under neutral conditions. (**C**) Nondenaturing PAGE analysis of **pH-DNG-ASO** assembly: (1) dendritic DNA, (2) linker, (3) shielding strand, (4) ASO strand, and (5) **pH-DNG-ASO**. (**D**) DLS spectrum of **pH-DNG, pH-DNG-ASO**, and **DOX@pH-DNG-ASO** (incubation of 1mM DOX). There is no significant differences (*p* > 0.05) in particle size among the groups pH-DNG vs. pH-DNG-ASO (*p* = 0.5071), pH-DNG vs. DOX@pH-DNG-ASO (*p* = 0.4219), and pH-DNG-ASO vs. DOX@pH-DNG-ASO (*p* = 0.9839). (**E**) Dynamic light scattering analysis of pH-DNG at pH 5.5 at different time points. (**F**) DOX release curve of **DOX@pH-DNG** at pH 7.4 and pH 5.5 for 24 h. Mean ± s.d., n = 3. (**G**) Nondenaturing PAGE analysis of ASO release from **pH-DNG-ASO** by target mRNA recognition, (1) **pH-DNG-ASO**, (2) **pH-DNG-ASO**+Target, (3) and (4) Target.

As depicted in Figure 1A, three crosslinking strands of dendritic DNA (**D**) contain a C-rich sequence that can form an i-motif under acidic pH [25] and hybridize to a G-rich sequence region of linker DNA (**L**) strands under neutral conditions. Furthermore, a 3′-palindromic tail of **L** induces intermolecular crosslinking and leads to the formation of pH-stimulus DNA nanohydrogels (**pH-DNG**). Atomic force microscopy (AFM) images revealed that **pH-DNG** is spherical in shape with an average diameter of approximately 68.04 ± 18.79 nm (Figure 1B) after air drying. Dynamic light scattering (DLS) measurements indicated an average hydrodynamic diameter of approximately 335.5 nm for **pH-DNG** in aqueous buffer (Figure 1D). Zeta potential showed **pH-DNG** exhibited a near-neutral surface charge of −7.345 mV, contrasting to that of D at −23.4 mV before nanohydrogel assembly (Figure S3). These characterizations demonstrated that D/L were successfully self-assembled into nanohydrogels with appropriate sizes as a nano-delivery vessel.

#### 3.1.2. Dual-Functional pH-DNG for Efficient Loading of Antisense Oligonucleotides (ASOs) and Chemotherapeutic Drugs

To fabricate an mRNA-responsive **pH-DNG**, we introduced an antisense RNA strand (ASO) of *HMGN5* to the loading strand of the dendritic DNA via a shielding strand (S) (Figure 1A). The loading was performed via a one-pot thermal annealing process, where dendritic oligonucleotides (D), linker strands (L), shielding strands (S), and ASO strands were mixed and heated to 95 °C, followed by slow cooling to room temperature. The resulting nanohydrogels were purified by dialysis to remove excess oligonucleotides and characterized by native polyacrylamide gel electrophoresis (PAGE), as shown in Figure 1C. The distinct band above 500 bp and slower mobility confirmed the successful formation of the ASO/pH dual-responsive DNG (**pH-DNG-ASO**). The size of **pH-DNG-ASO** was close to that of **pH-DNG**, with an average diameter of approximately 79.14 ± 18.01 nm in the dry state and 366 nm in aqueous buffer (Figure 1D and Figure S4). This observation indicates that the loading of ASOs does not disrupt the crosslinking of the hydrogel matrix, which could be ascribed to the spatial independence of the cross-linking and loading branches of dendritic DNA structure. Furthermore, when Cy5-labeled ASOs (Cy5-ASO) are used for assembly, a characteristic absorption peak at 650 nm (Figure S5) showed the efficient loading of Cy5-ASO onto **pH-DNG**. We next encapsulated the chemotherapeutic agent DOX into **pH-DNG** (**DOX@pH-DNG**) and **pH-DNG-ASO** (**DOX@pH-DNG-ASO**) according to our previous protocol [24]. Briefly, DOX was incubated with the nanohydrogel in a buffer solution (12.5 mM MgCl_2_, 1× TAE buffer, pH 7.4) at room temperature for 24 h in the dark. Unbound DOX was removed by dialysis, and the loading efficiency was quantified using UV–vis spectroscopy based on the extinction coefficient of DOX at 480 nm, as detailed in the Materials and Methods section. Figures S6A and S7 illustrate that both **pH-DNG** and **pH-DNG-ASO** achieved a high payload capacity of 323 DOX/bp. In addition, the hydrodynamic diameter of both DOX-loaded nanohydrogels showed negligible changes compared to **pH-DNG** (Figure 1D and Figure S6B).

#### 3.1.3. pH-Responsive Disassembly and Controlled Drug Release from pH-DNG

Next, pH-responsive behavior of **pH-DNG** was examined. DLS showed that the size of **pH-DNG** started decreasing after incubation at pH 5.5 for 5 min, and a majority of the nanohydrogels disintegrated into smaller particles below 25 nm and smaller entities below 1 nm over 6 h (Figure 1E). Furthermore, the surface charge of **pH-DNG** notably shifted to −18.9 mV after a 2 h incubation in an acidic environment (pH 5.5), suggesting that **pH-DNG** underwent degradation as crosslinking branches formed i-motif structures to break the hydrogel framework at pH 5.5 (Figure S3). Subsequently, the pH-responsive DOX release profile was assessed (Figure 1F). In pH 7.4 PBS, **DOX@pH-DNG** can retain up to 87% of drug molecules over 24 h. In contrast, at pH 5.5, nearly 70% of DOX was released. This difference indicates concurrent disassembly of **pH-DNG** with controlled DOX release in the acidic tumor microenvironment (TME), while drug leakage at physiological pH is minimal.

#### 3.1.4. Target-Specific Release of ASOs via Toehold-Mediated Strand Displacement

To assess ASO release, **pH-DNG-ASO** was incubated for 4 h with the target *HMGN5* mRNA and scrambled RNA, respectively. ASO delivered by **pH-DNG-ASO** is a DNA/RNA duplex with a toehold domain. Upon encountering a well-matched target mRNA, the DNA/RNA hybrids disassembled via a toehold-mediated strand displacement (TMSD) reaction [26]. Consequently, ASOs can bind to mRNA targets to facilitate gene interference. As illustrated by PAGE analysis (Figure 1G and Figure S8), a distinct newly emerged band could be observed in the presence of target mRNA, while no such new band was generated without target or scrambled mRNA, underscoring the high specificity of ASOs for their intended mRNA targets.

### 3.2. Biocompatibility and In Vitro Cytotoxic Effects of pH-DNG

Cytotoxicity assays were then used to assess the biocompatibility and therapeutic efficacy of nanohydrogels. Bare **pH-DNG** showed no obvious cytotoxicity up to 10 OD on both MDA-MB-231 and MCF-10A (Figure S9), indicating that DNA nanohydrogels have favorable biocompatibility as a safe drug delivery carrier. To initially validate the therapeutic potential of **pH-DNG**, we first evaluated its performance in A549 cells as a model system (Figure S10). A549 cells were treated with free DOX, **DOX@non-pH-DNG**, and **DOX@pH-DNG** for 48 h. The results revealed that while free DOX showed no apparent cytotoxicity at concentrations below 5 μM, the same dosage delivered by **pH-DNG** induced 72% cell death. Furthermore, at a concentration of 2 μM, **DOX@pH-DNG** exhibited a rapid increase in potency, reaching nearly 50% cytotoxicity, whereas **DOX@non-pH-DNG** showed only 17% cell death. These findings demonstrate that the cytotoxicity of doxorubicin was significantly enhanced by **pH-DNG**, which could be attributed to the efficient pH-responsive release of DOX in the acidic tumor microenvironment, ensuring sustained drug availability.

**Figure 2:**
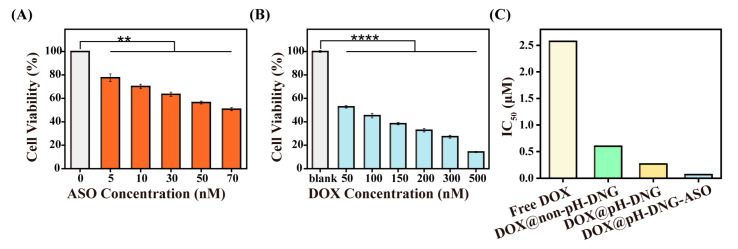
(**A**) Cell viability of MDA-MB-231 breast cancer cells after treatment with **pH-DNG-ASO** at different concentrations of ASOs for 48 h. (**B**) Cell viability of MDA-MB-231 treated with **DOX@pH-DNG-ASO** at different concentrations of DOX (the concentration of ASOs, 60 nM) for 48 h. (**C**) IC_50_ value of MDA-MB-231 treated with free DOX, **DOX@non-pH-DNG**, **DOX@pH-DNG**, and **DOX@pH-DNG-ASO** for 48 h. Asterisks represent significant differences and compared with blank control (** *p* < 0.01, **** *p* < 0.0001). Mean ± s.d., n = 3.

Subsequently, we treated MDA-MB-231 cells with free DOX, **DOX@non-pH-DNG**, **DOX@pH-DNG**, **pH-DNG-ASO**, **and DOX@pH-DNG-ASO**, respectively, for 48 h. **DOX@pH-DNG** demonstrated significantly greater cytotoxicity than both free DOX and **DOX@non-pH-DNG** in MDA-MB-231 cells (Figures S11 and S12), consistent with the enhanced efficacy observed in A549 cells. This consistency across different cancer types further validates the broad applicability of **pH-DNG** as a drug delivery platform. Additionally, **pH-DNG-ASO** displayed marked toxicity against MDA-MB-231 cells (Figure 2A). Therefore, the **DOX@pH-DNG-ASO** group exhibited the strongest inhibition of tumor cell proliferation (Figure 2B,C and Figure S12). While the IC_50_ of free DOX on MDA-MB-231 cells is 2.575 μM, the IC_50_ is reduced stepwise upon delivery by **non-pH-DNG**, **pH-DNG**, and **pH-DNG-ASO**. **DOX@pH-DNG-ASO** showed a near 40-fold decrease in IC_50_ to 0.06909 μM.

### 3.3. Efficient Cellular Internalization and Lysosomal Escape of pH-DNG-Delivered Therapeutics

**Figure 3:**
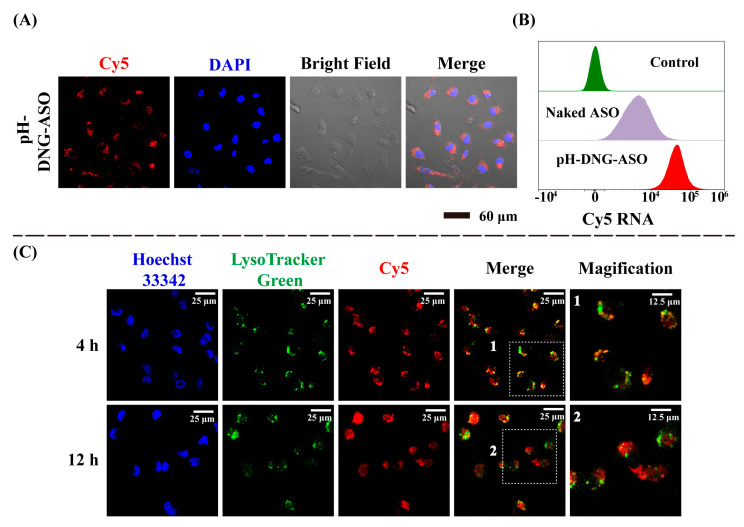
Cellular uptake and intracellular distribution of pH-DNG-ASO in MDA-MB-231 cells. (**A**) Confocal images of MDA-MB-231 cells after incubation with **pH-DNG-ASO** for 4 h. Cell nuclei were stained with DAPI (blue), and ASOs were labeled with Cy5 (red). (**B**) Flow cytometry analysis of cellular uptake of naked ASOs and **pH-DNG-ASO** (the concentration of ASOs, 60 nM) by MBA-MB-231 cells after incubation for 4 h. (**C**) Intracellular lysosomal escape of **pH-DNG-ASO** for 4 h, and 12 h. Cell nuclei were stained with Hoechst 33342 (blue), and lysosomes were labeled with Lysotracker Green (green).

To assess the internalization of DOX and ASOs via delivery by **pH-DNG**, we incubated MDA-MB-231 cells with fluorescent labeled **pH-DNG**, **DOX@pH-DNG**, **pH-DNG-ASO**, and **DOX@pH-DNG-ASO**, respectively. **pH-DNG** was assembled with TAMRA-labeled Linkers (TAMRA-L). ASOs were labeled with Cy5. Confocal fluorescent images (Figure 3A and Figures S13–S15) revealed that TAMRA-L (yellow), Cy5-ASOs (red), and DOX (green) fluorescence were significantly present in cells post incubation, indicating efficient intracellular delivery of DOX and ASOs by **pH-DNG**. Flow cytometry results also showed Cy5 fluorescence was more intense in the cell cohorts incubated with **pH-DNG-ASO** than those with free ASOs (Figure 3B). Moreover, intracellular Cy5 fluorescence increased progressively with extended incubation time of **pH-DNG-ASO**, demonstrating a time-dependent cellular internalization process (Figure S16).

To validate the lysosome escape capability of DNA nanohydrogels, MDA-MB-231 cells were incubated with **pH-DNG-Cy5-ASO** for 4 and 12 h. Cell nuclei were counterstained with Hoechst 33342 (blue) and the lysosomes were visualized with lysotracker Green (green). As shown in Figure 3C, almost all Cy5 and LysoTracker Green signals overlapped at 4 h as orange spots in merged images and indicated **pH-DNG-ASO** were within lysosomes to allow pH-triggered disintegration of nanohydrogels. After 12 h, Cy5 signals were observed outside LysoTracker Green foci, indicating the successful lysosomal escape of Cy5-ASOs into the cytoplasm for efficient targeting of mRNA. Colocalization analysis using ImageJ 1.54f software revealed a decrease in Pearson’s correlation coefficient from 0.954 to 0.775 over 8 h of incubation, further confirming the escape of **pH-DNG-ASO** from lysosomes into the cytoplasm.

### 3.4. Inhibition of Cell Migration and Downregulation of HMGN5 Expression

**Figure 4:**
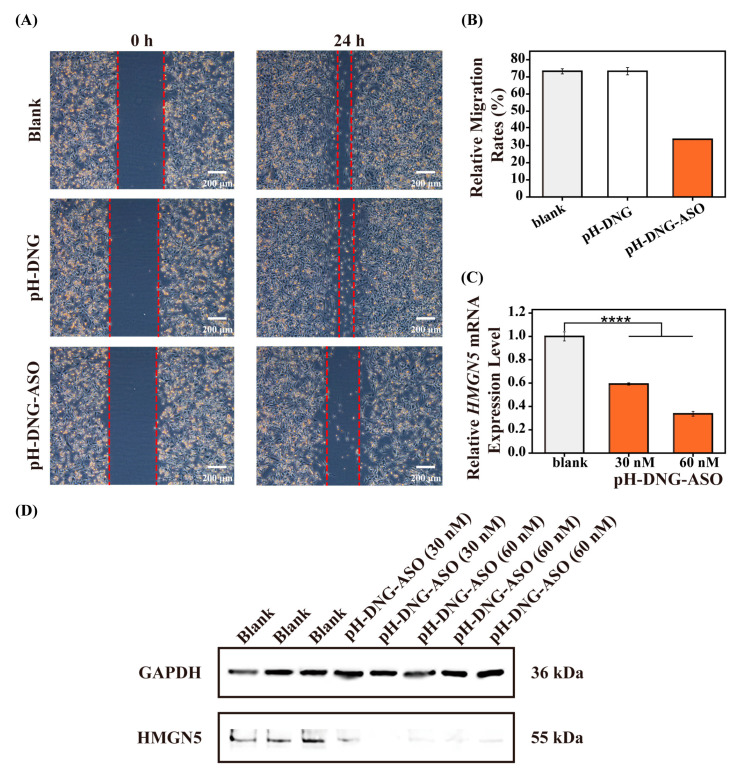
(**A**,**B**) Relative cell migration rate was determined using scratch healing assay in MDA-MB-231 cells incubated with PBS, **pH-DNG**, and **pH-DNG-ASO** (60 nM ASOs) for 24 h. (**C**) The gene expression level of *HMGN5* mRNA in MDA-MB-231 breast cancer cells after incubation with PBS and **pH-DNG-ASO** (30 nM and 60 nM, ASOs) for 48 h, respectively. Asterisks represent significant differences and compared with blank control (**** *p* < 0.0001). Mean ± s.d., n = 3. (**D**) Representative Western blot images showing HMGN5 and GAPDH (loading control) protein levels in MDA-MB-231 breast cancer cells after incubation with PBS, **pH-DNG-ASO** (30 nM), or **pH-DNG-ASO** (60 nM) for 48 h.

Additionally, to evaluate the efficiency of ASOs on downstream biological processes, we performed a scratch wound healing assay on MDA-MB-231 cells. MDA-MB-231 cells were subjected to a straight scratch and treated with PBS, **pH-DNG**, and **pH-DNG-ASO** (60 nM), respectively. As shown in Figure 4A,B, in both PBS and bare **pH-DNG** control groups, the scratch gap was nearly closed after 24 h. Conversely, the scratch in the **pH-DNG-ASO-**treated group remained open, suggesting that **pH-DNG-ASO** exerted a strong inhibitory effect on cell migration. We further quantified the expression level of *HMGN5* mRNA in MDA-MB-231 cells after incubation with **pH-DNG-ASO** for 48 h via qRT-PCR. Figure 4C showed that *HMGN5* mRNA downregulation was near 70% when cancer cells were incubated with **pH-DNG-ASO** (60 nM ASOs). Since the *HMGN5* gene is closely associated with the metastasis and proliferation of breast tumor cells, both assays showed an efficient inhibition of gene expression of *HMGN5* in terms of cell behavior and mRNA levels. To further validate the downregulation of HMGN5 at the protein level, Western blot analysis revealed a marked decrease in HMGN5 protein levels in cells treated with **pH-DNG-ASO** (60 nM) compared to the untreated control group (Figure 4D and Figure S17). These findings demonstrate that pH-DNG-ASO effectively inhibits HMGN5 expression at both the transcriptional and translational levels.

## 4. Discussion

Designing intelligent nanodelivery systems responsive to the tumor microenvironment (TME) is crucial for enhancing the precision and efficacy of cancer therapy. This study introduces a pH/ASO dual-responsive DNA nanohydrogel **(DOX@pH-DNG-ASO**) that integrates chemotherapeutic and gene therapy modules, demonstrating combined anticancer potential. The efficient drug loading and responsive mechanisms highlight the versatility of DNA nanomaterials in precision medicine.

**pH-DNG** forms spherical nanoparticles (~335 nm) with a near-neutral surface charge (−7.3 mV), optimizing tumor targeting via the enhanced permeability and retention (EPR) effect and minimizing nonspecific interactions. The modular design of dendritic DNA allows for ASO incorporation without disrupting the hydrogel matrix, enabling versatile cargo integration. The nanohydrogel rapidly disintegrates at pH 5.5 due to i-motif collapse, ensuring TME-specific drug release. DOX exhibits minimal leakage at physiological pH but rapid release in the acidic TME, maximizing accumulation at the target site while reducing off-target toxicity. This pH-dependent behavior leverages DNA hybridization for superior programmability compared to traditional carriers. ASO release is mediated by toehold-driven strand displacement, ensuring specificity for target *HMGN5* mRNA. This mechanism minimizes off-target effects, a common limitation of conventional antisense therapies. Efficient cytoplasmic delivery and lysosomal escape further enhance ASO functionality, overcoming biological barriers to nucleic acid delivery. The combined **DOX@pH-DNG-ASO** system reduces the IC_50_ by 40-fold compared to free DOX in MDA-MB-231 cells, attributed to TME-specific DOX accumulation and ASO-mediated HMGN5 silencing. Additionally, **pH-DNG** also enhanced DOX cytotoxicity in A549 cells, particularly at low drug concentrations. These results demonstrate the effectiveness of its pH-responsive release mechanism across different cancer types. In MDA-MB-231 cells, scratch assaying and qRT-PCR data show that ~70% *HMGN5* mRNA downregulation inhibits cancer cell migration, consistent with its role in breast cancer progression. Moreover, Western blot analysis verified that pH—DNG—ASO inhibited HMGN5 at the translational level by reducing its protein levels. In this combined treatment, gene therapy may enhance the efficacy of chemotherapy through the following mechanisms: For chemosensitization, knocking down *HMGN5* could impair DNA repair pathways such as homologous recombination, thereby potentiating DOX-induced DNA damage [27]. For drug resistance mitigation, *HMGN5* has been linked to multidrug resistance (MDR) through the regulation of ABC transporters, and its silencing may reverse chemoresistance [28].

Overall, this “chemo-gene” strategy enhances therapeutic efficacy while reducing required drug doses, aligning with the trends in combinatorial nanomedicine. However, the in vivo stability, biodistribution, and immune compatibility of **pH-DNG** require further validation. Future studies could explore broader mRNA targets, optimize ASO sequences, extend gene therapy applications to NSCLC-associated targets in A549 cells, test efficacy in additional cancer models, and evaluate clinical potential to advance **pH-DNG** as a universal combination platform.

## 5. Conclusions

In summary, we have successfully synthesized a multifunctional dendritic-DNA-based nanohydrogel that could efficiently deliver ASOs and DOX into breast cancer cells by precisely responding to the tumor microenvironment. The synthesis process of the nanocarrier was both convenient and highly efficient. Cell scratching and viability experiments showed that **DOX@pH-DNG-ASO** could not only inhibit cell migration but also effectively kill MDA-MB-231 cells. Lysosome escape, gene knock-down, and Western blot experiments revealed the underlying mechanism behind the dual treatments. Notably, **pH-DNG** enhanced DOX cytotoxicity in A549 cells, highlighting its potential as a universal drug delivery platform for diverse cancer applications. With these excellent advantages, this biocompatible DNA nanocarrier can be further rationally designed to load various components for more efficient combination treatments. In the future, we will investigate the efficacy of nanohydrogel on disease models with acidic microenvironments, such as triple-negative breast cancer (TNBC) and pancreatic ductal adenocarcinoma (PDAC), to leverage the pH-responsive properties of our system for targeted drug delivery in highly acidic tumor conditions. Thus, we envision that these well-organized DNA nanohydrogels can be applied for precise and efficient cancer theranostics and a broad range of bioengineering applications.

## Data Availability

The data supporting this article has been included as part of the ESI.

## Supplementary Materials

The following supporting information can be downloaded at: https://www.mdpi.com/article/10.3390/biom15040537/s1, Table S1: sequences of DNA and RNA; Figure S1: Scheme of chemical synthesis of dendritic DNA and assembly of pH-DNG. Figure S2: HPLC and Mass; Figure S3: Zeta potential; Figure S4: AFM, Figure S5: UV characterization; Figures S6 and S7: Encapsulation and calculation of DOX; Figure S8: Nondenaturing PAGE analysis; Figures S9–S12: Cell viability detection and IC_50_ calculation; Figures S13–S16: Cellular uptake analysis. Figure S17: Western blot image.
