# Supplementary material for: A pH-Responsive Dendritic-DNA-Based Nanohydrogel for Dual Drug Delivery
Source: Biomolecules. 2025 Apr 6;15(4):537. doi: 10.3390/biom15040537 (PMC12024947; doi:10.3390/biom15040537)
Supplement: Supplementary file 1 [file biomolecules-15-00537-s001.zip › biomolecules-3484983-supplementary.pdf]

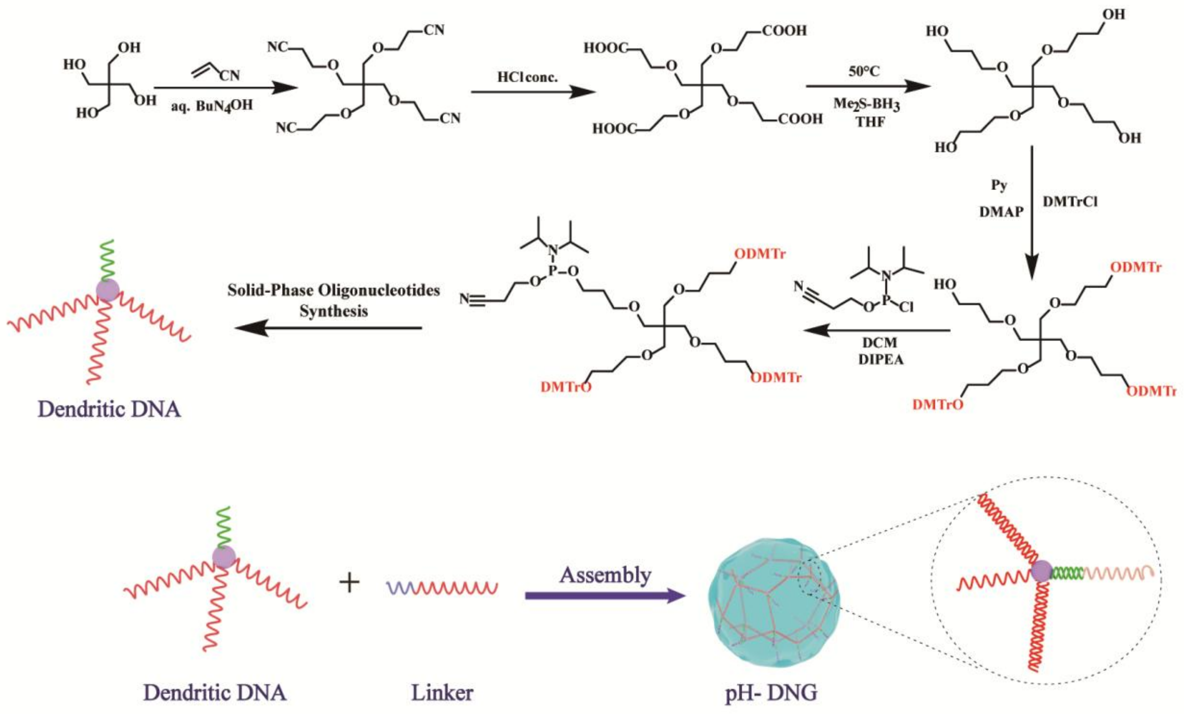

Figure S1. The chemical synthesis route of dendritic DNA and the schematic diagram of pH-DNA self-assembly.

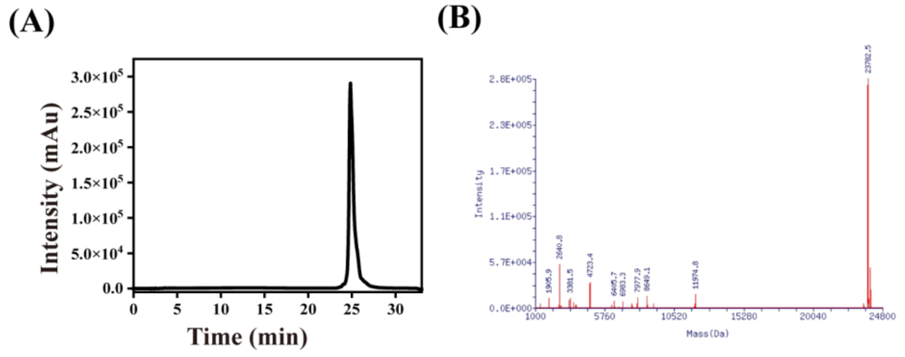

Figure S2. (A) The HPLC purification of dendritic DNA. (B) Mass of dendritic DNA.

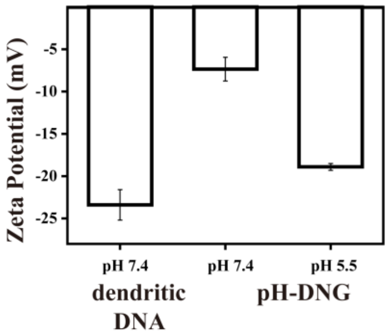

Figure S3. Zeta potential of dendritic DNA at pH 7.4, **pH-DNG** at pH 7.4 and pH 5.5.

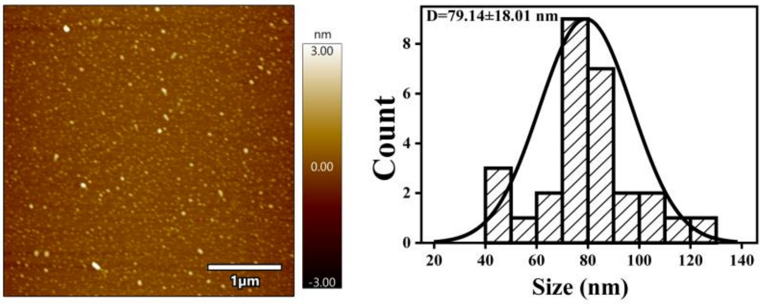

Figure S4. The AFM of **pH-DNG-ASO**.

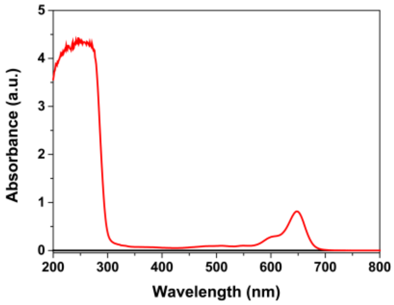

Figure S5. UV of **pH-DNG-ASO** (Cy5 labeled ASOs).

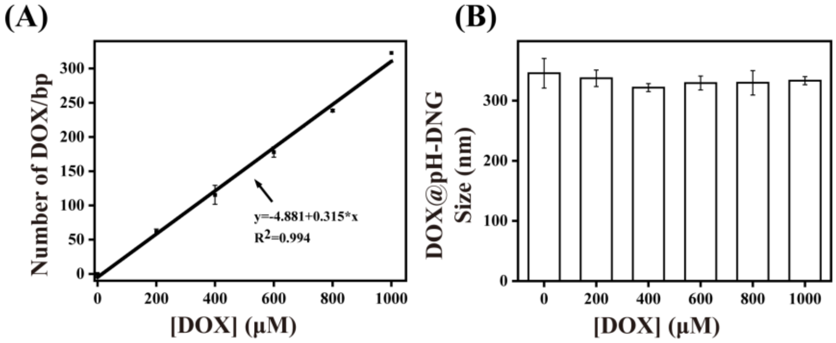

Figure S6. Encapsulation of DOX by **pH-DNG**. (A) Numbers of DOX per base pair loaded in **DOX@pH-DNG** after incubation with various concentrations of DOX. (B) The hydrodynamic sizes of **DOX@pH-DNG** after incubation with various concentrations of DOX.

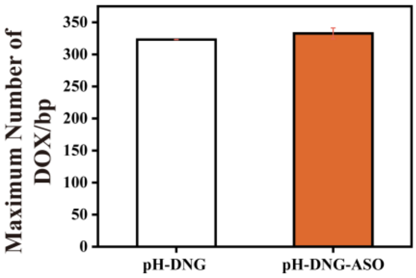

Figure S7. Maximum numbers of DOX per base pair loaded in **pH-DNG** and **pH-DNG-ASO** upon incubation of 1mM DOX.

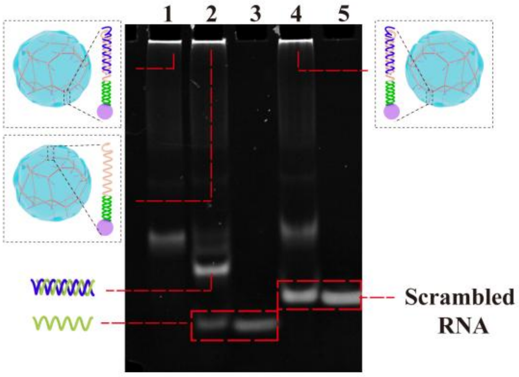

Figure S8. Nondenaturing PAGE analysis of **pH-DNG-ASO** incubated with target mRNA and scrambled RNA for 4 h. (1) **pH-DNG-ASO**, (2) **pH-DNG-ASO**+Target, (3) Target, (4) **pH-DNG-ASO**+scrambled RNA, (5) scrambled RNA.

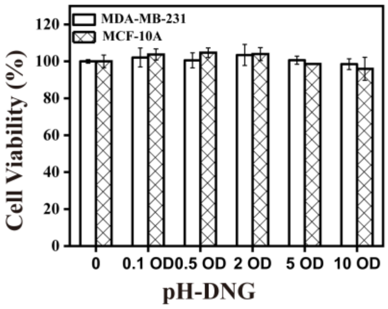

Figure S9. Cell viability of MDA-MB-231 and MCF-10A after incubation with **pH-DNG**.

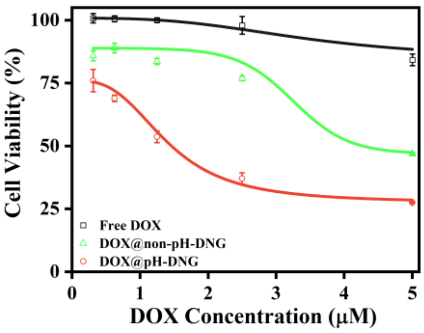

Figure S10. Cell viability of A549 cells after incubation with free DOX, **DOX@non-pH-DNG** and **DOX@pH-DNG** for 48 h. Mean ± s.d., n =3

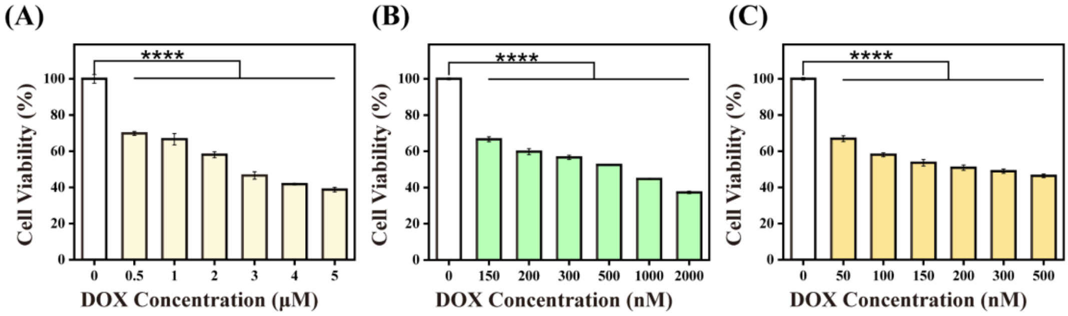

Figure S11. Cell viability of MDA-MB-231 treated with free DOX (A), **DOX@non-pH-DNG** (B) and **DOX@pH-DNG** (C) for 48 h. Asterisks represent significant differences (* P <0.05, ** P <0.01, *** P <0.001, **** P <0.0001). Mean ± s.d., n =3.

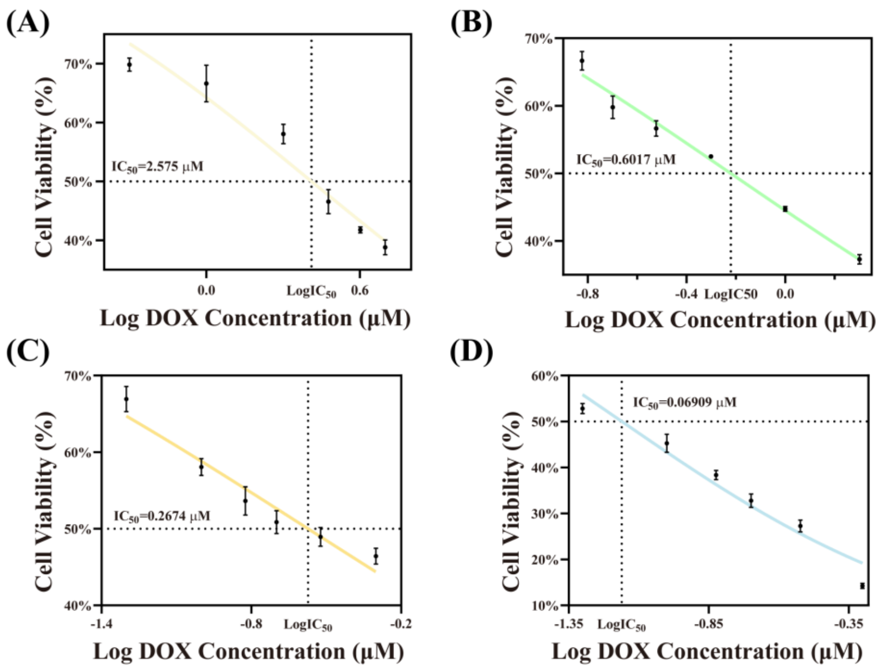

Figure S12. Calculate the IC_{50} value of MDA-MB-231 treated with free DOX (A), **DOX@non-pH-DNG** (B), **DOX@pH-DNG** (C), and **DOX@pH-DNG-ASO** (D) for 48 h using Graphpad. Mean ± s.d., n =3.

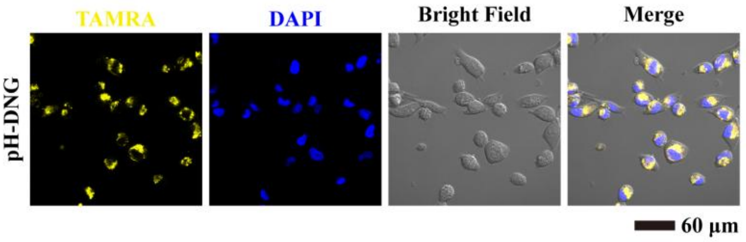

Figure S13. Cell uptake of **pH-DNG** by MDA-MB-231.

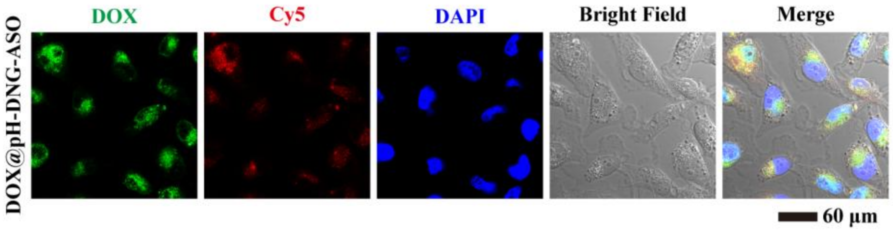

Figure S14. Cell uptake of **DOX@pH-DNG-ASO** by MDA-MB-231.

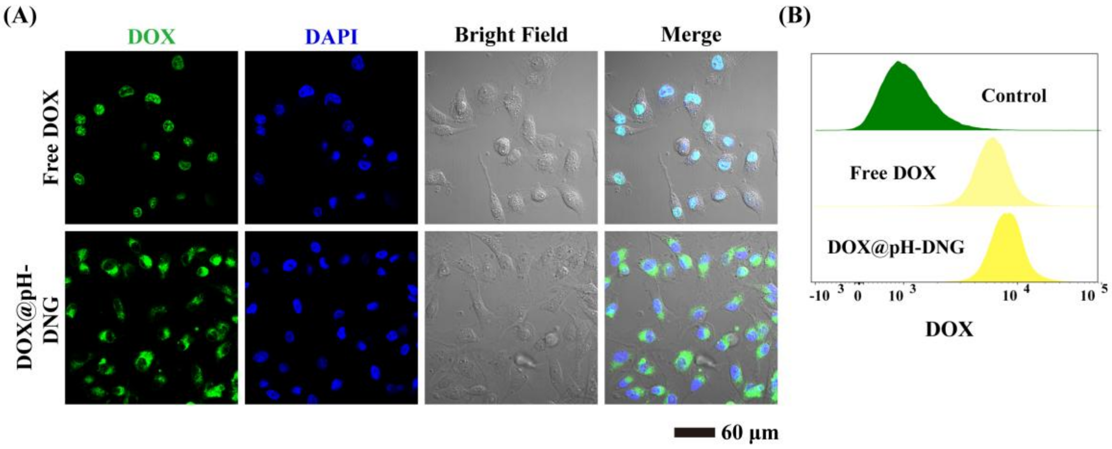

Figure S15. (A) Confocal images of MDA-MB-231 cells after incubation with free DOX and **DOX@pH-DNG** for 4 h, respectively. (B) Flow cytometry analysis of cellular uptake of free DOX and **DOX@pH-DNG** by MBA-MB-231 cells at 1 µM after incubation for 4 h.

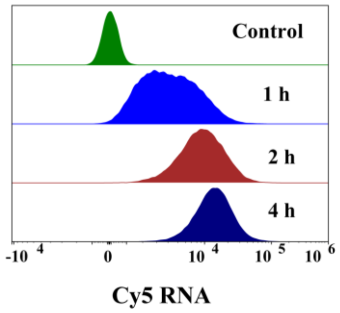

Figure S16. Flow cytometry analysis of cellular uptake of **pH-DNG-ASO** (the concentration of ASOs, 30 nM, Cy5 labeled on ASOs) by MBA-MB-231 cells at different time points after incubation.

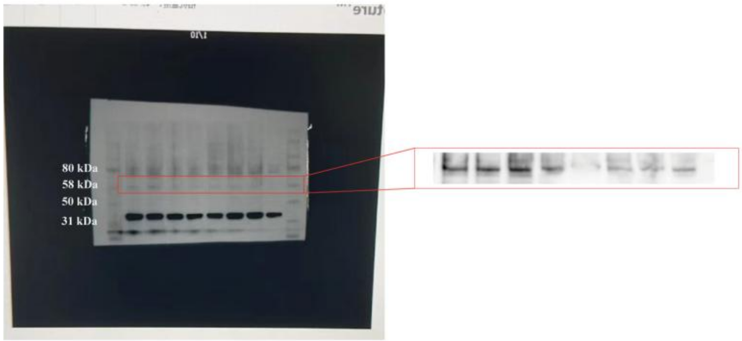

Figure S17. Full-length Western blot images of HMGN5 and GAPDH in MDA-MB-231 cells treated with PBS or **pH-DNG-ASO** (30 nM), or **pH-DNG-ASO** (60 nM) for 48 h. Molecular weight markers (kDa) are indicated on the left.

Table S1. DNA and RNA sequences.

DNA	Sequence (5' to 3')
Dendritic DNA	(CCC TAA CCC TAA CCC TAA CCC T)$_3$ D TTT CGA TCA TAG
Linker	AGG GTT AGG GTT AGG GTT AGG GAC AAC GTT GT
Linker-TAMRA	AGG GTT AGG GTT AGG GTT AGG GAC AAC GTT GT-TAMRA
Shielding strand	TTT AAG CCC AAG CAG TTT TTT CTA TGA TCG AAA
ASO	UUU CAG CAA CUG CUU GGG CTT
Cy5-ASO	Cy5-UUUCAGCAACUGCUUGGGCTT
Target mRNA	GCCCAAGCAGTTGCTGAAA
Scrambled RNA	UAGCUUAUCAGACUGAUGUUGA
HMGN5-Forward	CAGGTCAAGGTGATATGAGGCA
HMGN5-Reverse	GCTTGGGCACTTGTATCTATGT
GAPDH-Forward	GGAGCGAGATCCCTCCAAAAT
GAPDH-Reverse	GGCTGTTGTCATACTTCTCATGG
